# La convergencia de la ciencia de datos y la medicina de laboratorio

**DOI:** 10.1515/almed-2024-0133

**Published:** 2024-11-08

**Authors:** Javier Nieto-Moragas, Anna Marull Arnall, Fernando Calvo Boyero, Salomón Martin Pérez, Fernando Marqués García, Javier Hernando Redondo, Albert Blanco Grau, Cristian Cauqui Lende, Ángel Molina Borrás, Daniel Prieto Arribas, Elena de Rafael González

**Affiliations:** Grupo de Trabajo de Ciencia de Datos de la SEQC; Hospital Universitari de Bellvitge, Hospitalet de Llobregat, España; Hospital Universitari de Girona Dr Josep Trueta, Girona, España; Hospital Universitario 12 de Octubre, Madrid, España; Hospital Universitario Virgen Macarena, Sevilla, España; Hospital Germans Trias i Pujol, Badalona, España; Hospital del Mar, Barcelona, España; Hospital Universitari Vall d’Hebron, Barcelona, España; Hospital Universitario La Paz, Madrid, España; Complejo Hospitalario Universitario de Toledo, Toledo, España; Hospital Clínic de Barcelona, Barcelona, España

**Keywords:** machine learning, grupo de trabajo, ciencia de datos, formación

La ciencia de datos engloba el conocimiento para obtener y transformar datos con el fin de analizarlos y presentarlos tras verificar la calidad, robustez y su fácil interpretación. Estos conceptos son cotidianos en la actividad de los profesionales del laboratorio clínico así que los métodos computacionales y la consecución de procesos, conocidos como *pipelines*, presentan cierto paralelismo al manejo de muestras en los laboratorios. En el análisis de datos se utilizan diferentes herramientas para determinar su calidad y si es necesario adecuar su formato, en una fase de análisis se adecuan para su representación y modelización y finalmente se integra el resultado en un informe para su interpretación.

La convergencia entre la ciencia de datos y la medicina de laboratorio ha ido en aumento en los últimos años y ha suscitado gran interés entre los profesionales del laboratorio Clínico. Este interés no es casual, ya que en los laboratorios se trabaja con grandes dimensiones de datos de diferentes tipos y obtenidos mediante diferentes métodos analíticos. Por ello, las herramientas enmarcadas en la ciencia de datos tienen la capacidad de extraer información significativa de subconjuntos de datos complejos y de difícil comprensión si se analizaran de forma individual. El interés por integrar las aplicaciones de la ciencia de datos en los laboratorios se ha hecho presente en diversos países de una forma progresiva y a un mayor ritmo desde la pandemia del COVID-19.

A modo de ejemplo, podemos citar áreas de conocimiento que se engloban en la ciencia de datos y que se han vuelto cada vez más familiares recientemente como la inteligencia artificial, y el manejo de datos masivos, o *big data/data minig* en inglés. La aplicación de estas técnicas en el campo de la medicina permite el desarrollo de modelos de diagnóstico rápidos y precisos, una mejor elección de planes terapéuticos o la monitorización del paciente a tiempo real. En parte, estos hitos son posibles al empezar a trabajar con datos no estructurados (imágenes, audio o señales eléctricas) a tiempo real y que son fácilmente interpretables por la inteligencia artificial o explotables mediante *big data/data minig*. Los datos estructurados (en formato de tablas, listas o hojas de cálculo) también son de utilidad y han sido los más empleados hasta entonces de una forma retrospectiva junto con algoritmos de aprendizaje automático con el objetivo de optimizar tiempos de respuesta o facilitar la interpretación de los datos. A día de hoy, estas herramientas se agrupan bajo los conceptos de *Real World Data* (RWD) y *Real World Evidence* (RWE) que cada vez manejaremos con más frecuencia en nuestra actividad diaría [1].

En el siglo pasado, los especialistas en medicina del laboratorio comenzaron automatizando técnicas para mejorar el rendimiento y la reproducibilidad [2]. De forma progresiva, estos equipos han ido incorporando funcionalidades y manejan diferentes magnitudes, más allá de los resultados finales, como los datos demográficos, cinética de reacciones, alarmas, fluorescencia, cálculos, entre otros. Esta complejidad creciente obliga a otro gran cambio para su gestión, y los profesionales del laboratorio ya se han empezado a posicionar gracias a la formación complementaria, colaborando estrechamente con profesionales informáticos de su centro de trabajo o solicitando la implementación o solicitando a la industria del diagnóstico *in vitro* la implementación de mejoras basadas en nuevas tecnologías a la industria del diagnóstico *in vitro.* Con estas dinámicas se espera una revolución que transformará los sistemas sanitarios hacia una verdadera medicina personalizada y de precisión.

Los sistemas de información del laboratorio, disponibles en formato comercial con poca capacidad de personalización, introdujeron herramientas de gestión de datos en los laboratorios. Desde entonces han aparecido otras soluciones en formato de código abierto que incorporan mejoras continuas de forma ágil y permiten gestionar la información acorde a las necesidades de cada caso. Es fácil que el lector haya oído hablar de lenguajes de programación como Python, R o SQL, que se utilizan de forma creciente entre los profesionales para estadística descriptiva, cuadro de comandos o modelización de datos [3].

En el seno de la Sociedad Española de Medicina del Laboratorio (SEQC-ML), se ha impulsado la creación del nuevo Grupo de Trabajo en Ciencia de Datos que busca alcanzar los siguientes objetivos:

– Organizar actividades formativas y colaborar con otros profesionales para implementar la ciencia de datos en los laboratorios clínicos.
– Promover buenas prácticas para reducir posibles sesgos y errores en la implementación de las nuevas tecnologías con el respaldo de la industria del diagnóstico *in vitro*
– Garantizar una seguridad y trazabilidad de los datos siguiendo un marco legal y ético.

En este Grupo de Trabajo, se han incorporado profesionales con conocimientos avanzados en bases de datos, comunicación estandarizada e integración, sistemas de información, programación, modelización, entre otras, y de los que se espera que sirvan de referentes entre los demás compañeros. Algunos de los integrantes también forman parte de otras asociaciones, a nivel nacional e internacional, que también están comprometidas a impulsar el conocimiento relacionado con la Ciencia de Datos como la Federación de Asociaciones Científico Médicas Españolas (FACME) o la European Federation of Clinical Chemistry and Laboratory Medicine (EFLM).

Otras Sociedades Médicas, como la Sociedad Española de Nefrología o la Sociedad Española de Medicina Interna, han propuesto grupos de trabajo con objetivos parecidos a los del Grupo de Trabajo en Ciencia de Datos de la SEQC-ML y que avanzan para aplicar los conocimientos en inteligencia artificial o *big data/data minig* en sus respectivas áreas. Estas tecnologías son flexibles y a la par complejas, por lo que se evidencia la necesidad de incorporar expertos con conocimientos en ingeniería o matemáticas.

Se espera que los profesionales participen en la validación de modelos, transferencia de aplicaciones a la práctica asistencial y garantizar un marco ético y legal para la implementación de las soluciones tecnológicas con el fin de mejorar procesos de gestión de muestras o comunicación de resultados. Esta oportunidad eleva el valor de los profesionales de los laboratorios al requerir de una capacidad analítica y de creatividad y no se debe caer en el error de ver estas herramientas como una amenaza para el trabajo humano [4].

Cabe mencionar que el camino a recorrer presenta obstáculos que deben ser allanados por la Dirección de los centros de trabajo redirigiendo la dedicación en la digitalización o el *help desk* hacia la aplicación de nuevas técnicas que requieren de conocimientos médicos y presentan un valor añadido. De esta forma, los especialistas de los laboratorios con interés en estas nuevas tecnologías serán capaces de avanzar en la implementación de la ciencia de datos, siempre pensando en la utilidad para la práctica asistencial y aplicando sus conocimientos en medicina de laboratorio.

## References

[1] Ma C, Wang X, Wu J, Cheng X, Xia L, Xue F (2020). Real-world big-data studies in laboratory medicine: current status, application, and future considerations. Clin Biochem.

[2] Driscoll RC, Edwards RB, Liston MD, Love TW, Mazza JC, Trafton JE (1983). Discrete automated chemistry system with tableted reagents. Clin Chem.

[3] Mina A (2020). Big data and artificial intelligence in future patient management. How is it all started? Where are we at now? Quo tendimus?. Adv Lab Med.

[4] Cadamuro J (2021). Rise of the machines: the inevitable evolution of medicine and medical laboratories intertwining with artificial intelligence-A narrative review. Diagnostics.

